# Lung function and stress echocardiography in pulmonary arterial hypertension: a cross-sectional study

**DOI:** 10.1590/1516-3180.2021.0045.R1.0604221

**Published:** 2021-08-09

**Authors:** Guilherme Casagrande de Almeida, Mônica Corso Pereira, Marcos Mello Moreira, José Roberto Matos Souza, Ilma Aparecida Paschoal

**Affiliations:** I PT, MSc. Physiotherapist and Professor, Department of Physical Therapy, Paulista University, Campinas (SP), Brazil.; II MD, PhD. Professor, Discipline of Pulmonology, Department of Internal Medicine, School of Medical Sciences (FCM), University of Campinas (UNICAMP), Campinas (SP), Brazil.; III PT, PhD. Physiotherapist, Discipline of Pulmonology, Department of Internal Medicine, and Professor of Postgraduate Program on Surgical Sciences, School of Medical Sciences (FCM), University of Campinas (UNICAMP), Campinas (SP), Brazil.; IV MD, PhD. Professor, Discipline of Cardiology, Department of Internal Medicine, School of Medical Sciences (FCM), University of Campinas (UNICAMP), Campinas (SP), Brazil.; V MD, PhD. Full Professor, Discipline of Pulmonology, Department of Internal Medicine, School of Medical Sciences (FCM), University of Campinas (UNICAMP), Campinas (SP), Brazil.

**Keywords:** Hypertension, pulmonary, Capnography, Respiratory function test, Hypocapnia, Six-minute walk test, Arterial blood gas, Volumetric capnography, Lung function

## Abstract

**BACKGROUND:**

The mechanism of exercise limitation in idiopathic pulmonary arterial hypertension (IPAH) is not fully understood. The role of hemodynamic alterations is well recognized, but mechanical, ventilatory and gasometric factors may also contribute to reduction of exercise capacity in these individuals.

**OBJECTIVE:**

To investigate whether there is an association between ventilatory pattern and stress Doppler echocardiography (SDE) variables in IPAH patients.

**DESIGN AND SETTING:**

Single-center prospective study conducted in a Brazilian university hospital.

**METHODS:**

We included 14 stable IPAH patients and 14 age and sex-matched controls. Volumetric capnography (VCap), spirometry, six-minute walk test and SDE were performed on both the patients and the control subjects. Arterial blood gases were collected only from the patients. The IPAH patients and control subjects were compared with regard to the abovementioned variables.

**RESULTS:**

The mean age of the patients was 38.4 years, and 78.6% were women. The patients showed hypocapnia, and in spirometry 42.9% presented forced vital capacity (FVC) below the lower limit of normality. In VCap, IPAH patients had higher respiratory rates (RR) and lower elimination of CO_2_ in each breath. There was a significant correlation between reduced FVC and the magnitude of increases in tricuspid regurgitation velocity (TRV). In IPAH patients, VCap showed similar tidal volumes and a higher RR, which at least partially explained the hypocapnia.

**CONCLUSIONS:**

The patients with IPAH showed hypocapnia, probably related to their higher respiratory rate with preserved tidal volumes; FVC was reduced and this reduction was positively correlated with cardiac output.

## INTRODUCTION

Pulmonary arterial hypertension (PAH) is a rare disease characterized by increased pulmonary arterial pressure as a consequence of remodeling of arterial pulmonary microcirculation. Idiopathic pulmonary arterial hypertension (IPAH) is diagnosed after ruling out pulmonary hypertension associated with left heart disease, hypoxic lung diseases, chronic pulmonary thromboembolic disease and some other conditions of PAH.^1^

Clinically, IPAH produces severe and progressive limitation on physical exercise and activities of daily living. The exercise limitation is usually explained by the progressive reduction in cardiac output (CO), but there are still many gaps in the understanding of the interrelationships between hemodynamic abnormalities and respiratory mechanics. Volumetric capnography (VCap), spirometry and blood gas analysis can reveal aspects of ventilatory patterns that are not routinely investigated. We hypothesized that some of these resting variables could have correlations with hemodynamic variables collected during stress Doppler echocardiography (SDE).

## OBJECTIVE

In order to investigate this hypothesis, we sought to assess the resting breathing pattern among patients with IPAH, by means of VCap, arterial blood gases and spirometry. Patients were also evaluated during exercise by means of the six-minute walk test (6MWT) and SDE. We then sought to identify whether the variables obtained at rest would correlate with those collected during exercise.

## METHODS

This was a single-center prospective observational study conducted at the Department of Pulmonology of a Brazilian university hospital. We considered for inclusion all patients with IPAH, of both sexes and over 18 years of age, who met the hemodynamic and clinical criteria stated in the latest PAH guidelines.^1^ After reviewing the medical records, patients who fulfilled the inclusion criteria of the New York Heart Association (NYHA) functional class I-III and oxygen saturation (SpO_2_) ≥ 90% were invited to participate. We excluded smokers (and ex-smokers), individuals who presented asthma or cardiac diseases and individuals who were unable to perform the tests. Inclusion of patients and data collection occurred between January 2011 and June 2015. The control group consisted of healthy non-smoker volunteers who were matched for age and sex with the group of patients, with no history or current heart or respiratory disease and with no regular physical training done.

This study was approved by a local research ethics committee (ruling 1129/2010; November 23, 2010). All the patients participating in the study signed a written informed consent statement.

All the subjects answered a questionnaire that asked about symptoms, medications used and functional class of dyspnea (NYHA). We also calculated their body mass index (BMI) and measured SpO_2_ at rest. The tests were performed in the same sequence, with 15 minutes between them, and included VCap, blood sampling for gas analysis (only in patients), spirometry, 6MWT and exercise SDE.

VCap was performed using a CO_2_SMOS Plus 8100 device (Dixtal/Novametrix Respironics, Murrysville, Pennsylvania, United States). The subjects remained breathing tidal volume for five minutes. During this time, the variables were measured and data was stored in a computer equipped with the Analysis Plus software 1996 (Respironics, Murrysville, Pennsylvania, United States). At the end of data collection, an offline sequence from the respiratory cycles of each subject was selected to accommodate variation of 15% for expiratory tidal volume and 5% for partial end-tidal CO_2_ (EtCO_2_) tension. Respiratory cycles that had slope 2 (Slp2) and slope 3 (Slp3) equal to zero were excluded.^2^^,^^3^ The main variables analyzed were EtCO_2_, Slp2, Slp3, inspiratory time (Ti), expiratory time (Te), expiratory volume (Ve) and tidal volume according to anatomical dead space (Vd/Vt aw). Both slopes were calculated using the Analysis Plus software.

Spirometry was performed (EasyOne-PC, NDD Medizintechnik AG, Zurich, Switzerland) in accordance with the Brazilian guidelines and reference values for the Brazilian population were used.^4^ Values for forced vital capacity (FVC), forced expiratory volume in one second (FEV_1_) and FEV_1_/FVC ratio were analyzed.

All subjects performed the 6MWT under supervision by the same technician, in accordance with the American Thoracic Society guidelines.^5^ Baseline blood pressure and heart rate (HR) were measured, and SpO_2_ was determined using a finger probe pulse oximeter (3100 Wrist-Ox wrist pulse oximeter; Nonin Medical, Plymouth, Minnesota, United States), three times: at rest, in the sixth minute (end of the test) and in the ninth minute (recovery). During the test, the patients were carefully observed to ensure that their exercise limits would not be dangerously exceeded. The distance was measured in meters and desaturation (∆SpO_2_) was calculated as follows: SpO_2_ in the sixth minute minus initial SpO_2_.

For stress echocardiography, a variable-load supine exercise bicycle [Movement 4000; Movement, São Paulo (SP), Brazil] was used and the test was always performed by the same physician (JRM) using a 3 mHz probe (Xario PST-30BT; Toshiba, Kyoto, Japan). The workload was increased by 25 W every two minutes until the submaximal HR was reached (85% of the predicted maximum HR), or the symptom-limited maximum. HR, SpO_2_ and tricuspid regurgitation velocity (TRV) were analyzed twice: at rest and during stress. Systolic pulmonary arterial pressure (SPAP) was estimated from peak TRVs in accordance with the following equation: SPAP = 4(V)^2^ + right atrial pressure, where V is the peak velocity (in m/s) of TRV. Pulmonary vascular resistance (PVR) was estimated using the formula: PVR = (simple ratio of peak tricuspid regurgitation velocity/right ventricular outflow tract velocity-time integral) x 10 + 0.16.^6^ Cardiac output (CO) was determined using the following equation: CO = (systolic volume x HR)/1000.^7^ The variations between rest (r) and stress (s) were calculated for TRV, SPAP, PVR and CO and were designated as ∆TRV = TRV_s_ – TRV_r_; ∆SPAP = SPAP_s_ – SPAP_r_; ∆PVR = PVR_s_ – PVR_r_; and ∆CO = CO_s_ – CO_r_, respectively. Echocardiographic assessments were performed in accordance with current guidelines.^8^

### Data analysis

Exploratory data analysis was performed using summary measurements (mean, standard deviation, minimum, median, maximum, frequency and percentage). The groups were compared using the Mann-Whitney test or Fisher’s exact test, as appropriate. The factors for ∆TRV and ∆CO were evaluated by means of linear regression, after transformation of the variables into ranks. Probability values of less than 0.05 were considered to be statistically significant. The analysis were performed using the SAS System for Windows (Statistical Analysis System, version 9.4; SAS Institute Inc., Cary, North Carolina, United States).

## RESULTS

We evaluated the medical records of 99 patients with a previous diagnosis of IPAH who were being followed at our outpatient clinic. Of these, 24 met the inclusion criteria, but 10 declined to participate, leaving 14 patients who completed all the proposed tests. Fourteen control subjects who were matched for age and sex with the group of patients were also enrolled. The clinical data are shown in **Table 1**, and there were no differences between the groups regarding age, sex or BMI. None of the subjects in the control group had any respiratory symptoms.

**Table 1.** Clinical data on idiopathic pulmonary arterial hypertension (IPAH) patients and control subjects

		IPAH group n = 14	Control group n = 14	P-value
Age (years)^*^		38.4 ± 9.1	38.5 ± 9.1	0.85
Sex (women, n/%)		11 (78.6%)	11 (78.6%)	1.00
Body mass index (kg/m^2^)^*^		24.0 ± 3.6	24.8 ± 23.6	0.66
New York Heart	I	0 (0.0%)	14 (100%)	
Association functional	II	9 (64.3%)	0 (0.0%)
class (n/%)	III	5 (35.7%)	0 (0.0%)	

^*^ Values expressed as mean ± standard deviation.

Data on lung function and functional capacity (6MWT) are shown in **Table 2.** In spirometry, the IPAH patients had lower FVC and lower FEV_1_ than the control subjects.

**Table 2.** Functional variables of idiopathic pulmonary arterial hypertension (IPAH) patients and control subjects

		IPAH group n = 14	Control group n = 14	P-value
**Spirometry**	FVC (liters)	3.0 ± 0.6	3.7 ± 0.6	0.018
	FEV_1_ (liters)	2.5 ± 0.5	3.0 ± 0.5	0.017
	FEV_1_/FVC (%)	81.5 ± 4.4	81.6 ± 6.9	0.35
	FVC < LLN (n/%)	6 (42.9%)	0 (0.0%)	0.016
**Volumetric capnography (VCap)**	RR (cycles/minute)	16.9 ± 4.2	13.4 ± 4.3	0.020
	Vd/Vt aw	0.3 ± 0.0	0.3 ± 0.1	0.40
	VCO_2_ (mmHg)	178.4 ± 47.5	190.5 ± 56.9	0.066
	Ve (ml)	532.9 ± 151.3	643.4 ± 288.8	0.37
	T_i_ (minutes)	1.5 ± 0.4	2.0 ± 0.7	0.037
	T_e_ (minutes)	2.3 ± 0.8	3.0 ± 1.0	0.020
	ETCO_2_	29.4 ± 8.1	33.9 ± 4.7	0.069
	VCO_2_/br (mmHg/br)	10.6 ± 3.3	16.4 ± 7.7	0.012
	Slp3/Ve (mmHg/l)	0.0 ± 0.1	0.0 ± 0.0	0.87
**Arterial blood**	PaCO_2_ (mmHg)	29.5 ± 4.6	-	-
**Six-minute walk test (6MWT)**	Borg at rest	1.4 ± 2.1	0.0 ± 0.1	0.023
	Borg end	4.4 ± 2.9	0.2 ± 0.5	< 0.0001
	HR at rest	84.1 ± 16.1	88.0 ± 14.9	0.45
	HR end	130.5 ± 24.7	125.9 ± 19.4	0.70
	SpO_2_ at rest (%)	97.6 ± 1.7	95.4 ± 4.2	0.38
	∆SpO_2_ (%)	-3.9 ± 5.8	-0.9 ± 1.3	0.089
	6MWT (meters)	424.4 ± 139.4	595.1 ± 54.6	0.0001

Values expressed as mean ± standard deviation. FVC = forced vital capacity; FEV_1 =_ forced expiratory volume in one second; LLN = lower limit of normality; RR = respiratory rate during VCap; Vd/Vt aw = ratio of tidal volume to anatomical dead space; VCO_2 =_ excretion of carbon dioxide; Ve = expiratory volume; Ti = inspiratory time; Te = expiratory time; EtCO_2_ = end-tidal CO_2_; VCO_2_/br = excretion of CO_2_ per respiratory cycle; Slp3/Ve = slope 3 normalized according to expired volume; PaCO_2_ = partial pressure of carbon dioxide in the arterial blood; Borg = scale for evaluation of the degree of respiratory discomfort before (at rest) and at the end of 6MWT test; HR = heart rate; SpO_2 =_ oxygen saturation of hemoglobin; ∆SpO_2 =_ SpO_2_ in the sixth minute minus initial SpO_2_; 6MWT = six-minute walk test.

In the VCap evaluation, the IPAH patients had lower values than the control group, in relation to VCO_2_/br, Ti and Te. In addition, the respiratory rate was higher than in the control group. In blood gas analysis, all the IPAH patients presented hypocapnia at rest (PaCO_2_ = 29.5 ± 4.6 mmHg).

In the 6MWT, the IPAH patients had significantly higher Borg index values before and at the end of the test, compared with the control group. They also walked shorter distances than the control group.

The stress Doppler echocardiography (SDE) results are shown in **Table 3**. All the IPAH patients discontinued the SDE test due to physical exhaustion, but all had a HR above 85% of maximal HR (220 beats per minute minus the patient’s age). In the control group, the test was halted when submaximal HR was reached (above 80% of maximal HR).

**Table 3.** Stress Doppler echocardiography (SDE) variables of IPAH patients and control subjects

			IPAH group n = 14	Control group n = 14	P-value
**Stress Doppler echocardiography (SDE)**
	TRV (meter/second)	TRV at rest	3.5 ± 0.9	1.8 ± 0.2	< 0.0001
		TRV max	4.9 ± 1.2	2.4 ± 0.2	< 0.0001
		∆TRV	1.4 ± 0.6	0.6 ± 0.3	0.0001
	PVR (dyn.s/cm^5^)	PVR at rest	2.4 ± 0.9	1.3 ± 0.2	0.0003
		PVR max	3.2 ± 1.4	1.2 ± 0.5	< 0.0001
		∆PVR	0.8 ± 1.0	-0.1 ± 0.5	0.011
	CO (ml/min)	CO at rest	3124.2 ± 989.1	3903.6 ± 980.3	0.094
		CO max	6989.0 ± 3063.8	11780.8 ± 2616.6	0.0002
		∆CO	3864.8 ± 2605.0	7877.1 ± 2202.1	0.0004
	SPAP (mmHg)	SPAP at rest	61.9 ± 28.6	23.6 ± 2.7	< 0.0001
		SPAP max	109.3 ± 44.6	33.8 ± 4.4	< 0.0001
		∆SPAP	47.4 ± 26.9	10.2 ± 4.8	< 0.0001

TRV = tricuspid regurgitation peak velocity; PVR = pulmonary vascular resistance; CO = cardiac output; SPAP = systolic pulmonary arterial pressure; ∆PVR = PVR under stress – PVR at rest; ∆SPAP = SPAP under stress – SPAP at rest; ∆CO = CO under stress – CO at rest.

The IPAH patients presented higher values for TRV than the control patients at rest (TRVr) and at exercise peak (TRVs). In addition, ∆TRV was higher in the patient group. Also, higher values for pulmonary vascular resistance and SPAP were observed in the IPAH group, compared with the control subjects. The two groups presented similar CO values at rest, but CO under stress and the difference between stress and rest (∆CO) were significantly lower in the patient group than in the control group.

In the correlation analysis, there were positive correlations between ∆TRV and FVC, pulmonary vascular resistance under stress (PVRs) and the variation in PVR during SDE (∆PVR). Thus, the higher the values of ∆TRV were, the higher the values of PVR, ∆PVR and FVC also were. We also found positive correlations between ∆CO and the values for BMI and PaCO_2_; and a negative correlation between ∆CO and Vd/Vt aw. (**Table 4**)

**Table 4.** Factors associated with variation of tricuspid regurgitation peak velocity (TRV) and cardiac output (CO) during stress Doppler echocardiography (SDE)

	R	Factors analyzed	P-value
∆TRV	Positive correlation	∆PVR	0.0354
	Positive correlation	PVRmax	0.0470
	Positive correlation	FVC	0.0293
∆CO	Positive correlation	BMI	0.0005
	Positive correlation	PaCO_2_	0.0024
	Negative correlation	Vd/Vt aw	0.0234

∆TRV = change in TRV during SDE; ∆CO = change in CO during SDE; PVR = pulmonary vascular resistance; ∆PVR = change in PVR during SDE; PVR max = pulmonary vascular resistance under stress; FVC = forced vital capacity; BMI = body mass index; PaCO_2 =_ partial pressure of carbon dioxide; Vd/Vt aw = ratio of tidal volume to anatomical dead space during the evaluation of VCap.

## DISCUSSION

The patients presented hypocapnia at rest and, in comparison with the control subjects, had lower FVC. A higher proportion of the patients had FVC below the lower limit of normality (42.9% versus 0%), even with FEV_1_/FVC ratio > 0.8. In VCap, the IPAH patients presented a higher respiratory rate than the control subjects, with similar tidal volumes (Vi and Ve). Also, the VCO_2_ eliminated at each expiration was lower in the patients. Taken together, these findings suggest a respiratory pattern at rest indicative of restrictive disorder and tachypnea.

The low PaCO_2_ and higher respiratory rates measured in VCap suggested that the IPAH patients were hyperventilating even during rest, which could explain the hypocapnia. In fact, there is evidence that patients with PAH seem to hyperventilate during exercise, at rest and even during sleep.^9^ Some authors found hypocapnia (PaCO_2_ < 35 mmHg) in patients with pulmonary hypertension^10^^,^^11^ and Hoeper et al. also showed that in IPAH patients, hypocapnia seemed to be an independent marker for mortality, whereas PaO_2_ had no significant prognostic value.^10^

Patients with left heart failure and pulmonary hypertension (PH) have low cardiac outputs, but the effects of these conditions on the lungs are very different. The consequences of low CO for left heart failure are significant for peripheral organs and tissues, but the lungs are filled with fluid. Lungs with pulmonary edema have reduced compliance, and as expected from the mathematically inverse relationship, increased elastance.^12^ In contrast, patients with PAH have less fluid in their lungs, as a result of remodeling of pulmonary microcirculation and increased pulmonary resistance. The reduction of the total amount of blood in the lungs of these individuals increases compliance and can make lung expansion easier. Increased compliance facilitates lung expansion and may alter the mechanisms of early discontinuation of lung expansion during inspiration. Along these lines, in a study on dynamic hyperinflation during exercise among patients with precapillary pulmonary hypertension, Richter et al. found a weak negative correlation between the change in inspiratory capacity and pulmonary vascular resistance.^11^

In spirometry, our patients had lower FVC and FEV_1_ and a higher proportion of these individuals had FVC below the lower limit of normality, compared with the control group. These findings are similar to what was found by other authors.^11^^,^^13^^,^^14^ Meyer et al. evaluated lung function in patients with pulmonary hypertension (PH), compared with controls, and they identified signs of peripheral airway obstruction, seen as reductions in FVC and FEV_1_/FVC, and increases in residual volume and the residual volume/total lung capacity ratio.^14^ These authors postulated that there was evidence of premature airway closure, leading to reduction in FVC, perhaps due to impairment of lung elastic recoil.

In our study, we also found a positive correlation between ∆TRV (difference in tricuspid regurgitation velocity between rest and peak effort) and FVC (P = 0.029). Richter et al. described a negative correlation between the change in inspiratory capacity (IC) during exercise and the pulmonary vascular resistance. Moreover, they observed that the reduction in IC seemed to be related to a decrease in aerobic exercise capacity.^11^ The same authors suggested that IC might have prognostic value among patients with PH: patients with better IC at rest (> 89% of predicted values) have significantly better survival rates than those with IC ≤ 89%.^15^

Laveneziana et al.^16^ also identified signs of dynamic hyperinflation (DH) and reduction of IC as factors involved in exercise limitation among PAH patients.

Meyer et al. reported that residual volumes and the residual volume/total lung capacity ratios were significantly higher in PH patients than in controls, but that airway resistance was similar in the two groups. Those patients showed airflow limitation that could be explained by loss of elastic recoil.^14^

Considering that the reduction in FVC may be related to the reduction in resting inspiratory capacity, it can be speculated that this reduction of FVC may have some prognostic significance. The positive correlation between ∆TRV and FVC that we found in our study gives strength to this hypothesis, since exercise-induced increases in TRV and PSAP may be considered to be measurements of right ventricular contractile reserve. Using stress Doppler echocardiography, Grünig et al. demonstrated that exercise-induced increases in PSAP had clinical and prognostic relevance in PH patients, such that the lower the pressure increase was, the worse the prognosis also was.^17^

VCap evaluation showed that the IPAH patients had increased respiratory rates even at rest, without changes in expired volumes. Interestingly, they eliminated less CO_2_ per breath (P = 0.012) and had lower EtCO_2_, although this difference was not statistically significant (P = 0.069). Some investigators have shown that patients with PAH have low EtCO_2_, and that they present a further decrease from baseline in cardiopulmonary exercise testing.^18^-^20^

As discussed above, the patients in this study had hypocapnia at rest, a condition that explains their low CO_2_ values per breath (VCO_2_/br) and low EtCO_2_. There was no significant difference in the elimination of CO_2_ per minute between the cases and controls, given that the patients had higher respiratory rates.

It is also noteworthy that CO_2_ elimination is related to CO, and that VCap has been used to monitor the efficiency of cardiac resuscitation procedures. However, this relationship may not be linear. Smaller reductions in CO than those observed in cardiac arrest are probably not detected through VCap.

Although our results are tentative, they suggest that there is a characteristic ventilatory pattern in patients with IPAH. The reduction in FVC, the hypocapnia and the VCap findings might be associated with reduction in pulmonary perfusion and low right ventricular contractile reserve.

### Limitations and strengths

One of the strengths of our study is that it searched for noninvasive methods for assessing patients with PH, such as VCap. Lung function has been poorly studied in patients with PH, and findings such as reduced vital capacity or hypocapnia may have clinical relevance as biomarkers. Considering the small number of patients studied here, the findings cannot be generalized to all patients with pulmonary hypertension. The unavailability of lung compliance and elastance analysis prevented confirmation of the hypothesis raised in this study. Our findings are tentative and need further investigation. Nevertheless, the idea is appealing and, from our perspective, deserves consideration.

## CONCLUSIONS

Patients with IPAH showed hypocapnia, reduced FVC and reduced elimination of CO_2_. These features could be explained as consequences of reduction in lung perfusion, which is a typical finding in IPAH.
